# Numerous *Fasciola* plasminogen-binding proteins may underlie blood-brain barrier leakage and explain neurological disorder complexity and heterogeneity in the acute and chronic phases of human fascioliasis

**DOI:** 10.1017/S0031182018001464

**Published:** 2018-09-24

**Authors:** J. González-Miguel, M. A. Valero, M. Reguera-Gomez, C. Mas-Bargues, M. D. Bargues, F. Simón, S. Mas-Coma

**Affiliations:** 1 Laboratorio de Parasitología, Instituto de Recursos Naturales y Agrobiología de Salamanca (IRNASA-CSIC), Cordel de Merinas 40-52, 37008 Salamanca, Spain; 2 Departamento de Parasitología, Facultad de Farmacia, Universidad de Valencia, Av. Vicent Andrés Estellés s/n, 46100 Burjassot, Valencia, Spain; 3 Departamento de Fisiología, Facultad de Medicina, Universidad de Valencia, Av. Blasco Ibáñez No. 15, 46010 Valencia, Spain; 4 Área de Parasitología, Facultad de Farmacia, Universidad de Salamanca, Av. Licenciado Méndez Nieto s/n, 37007 Salamanca, Spain

**Keywords:** Acute and chronic phases, blood-brain barrier leakage, contact system, *Fasciola* excretome/secretome, fibrinolysis system, human fascioliasis, indicators and prevention, neurological disorders, plasminogen-binding proteins, proteomic and mass spectrometry analyses

## Abstract

Human fascioliasis is a worldwide, pathogenic food-borne trematodiasis. Impressive clinical pictures comprising puzzling polymorphisms, manifestation multifocality, disease evolution changes, sequelae and mortality, have been reported in patients presenting with neurological, meningeal, neuropsychic and ocular disorders caused at distance by flukes infecting the liver. Proteomic and mass spectrometry analyses of the *Fasciola hepatica* excretome/secretome identified numerous, several new, plasminogen-binding proteins enhancing plasmin generation. This may underlie blood-brain barrier leakage whether by many simultaneously migrating, small-sized juvenile flukes in the acute phase, or by breakage of encapsulating formations triggered by single worm tracks in the chronic phase. Blood-brain barrier leakages may subsequently occur due to a fibrinolytic system-dependent mechanism involving plasmin-dependent generation of the proinflammatory peptide bradykinin and activation of bradykinin B2 receptors, after different plasminogen-binding protein agglomeration waves. Interactions between diverse parasitic situations and non-imbalancing fibrinolysis system alterations are for the first time proposed that explain the complexity, heterogeneity and timely variations of neurological disorders. Additionally, inflammation and dilation of blood vessels may be due to contact system–dependent generation bradykinin. This baseline allows for search of indicators to detect neurological risk in fascioliasis patients and experimental work on antifibrinolytic treatments or B2 receptor antagonists for preventing blood-brain barrier leakage.

## Introduction

Fascioliasis is a worldwide food-borne trematodiasis caused by two *Fasciola* species transmitted by freshwater lymnaeid snails: *Fasciola hepatica* in Europe, Africa, Asia, the Americas and Oceania, and *Fasciola gigantica* in parts of Africa and Asia (Mas-Coma *et al*., 2009). This disease is well known in the veterinary field due to the economic losses it causes in animal husbandry including livestock morbidity and mortality (Torgerson and Claxton, 1999). A new serious public health scenario progressively appeared from the 1990s (Chen and Mott, 1990), with the description of many human endemic areas and non-stop increasing number of case reports (Mas-Coma *et al*., 2014*a*). This led to demonstrate that it is the vector-borne parasitic disease with the widest latitudinal, longitudinal and altitudinal distribution known and to reach a global estimation of 17 million people infected (Mas-Coma, 2005).

This new scenario emphasizes the importance of the disease chronic phase, hitherto considered secondary when compared with the acute phase. Indeed, almost all (if not all) subjects diagnosed in human endemic areas are in the chronic stage (Mas-Coma *et al*., 2014*a*), whereas in developed countries many or most patients are diagnosed in the acute phase (Arjona *et al*., 1995). Efforts to go deep in the advanced chronic phase demonstrated its implications in pathogenicity (Valero *et al*., 2003, 2006, 2008, 2016), immunogenicity (Girones *et al*., 2007) and in the reinfections during this chronic period (Valero *et al*., 2017).

A recent analysis has proved that a very wide range of neurological, meningeal, neuropsychic and ocular disorders caused at distance by flukes infecting the liver may be relatively frequent but underestimated due to misdiagnosis, mainly in low-income regions. The impressive clinical pictures comprise puzzling polymorphisms, disconcerting multifocality of the manifestations, changes along the disease evolution in a patient and differences between the clinical pictures shown by different patients. In these patients, moreover, post-treatment sequelae and mortality should be emphasized (Mas-Coma *et al*., 2014*b*). No mechanism able to explain the triggering of neurological, meningeal and/or ophthalmic disorders at distance by flukes present in the liver or neighbouring tissues has been so far proposed.

Although the molecular and biochemical mechanisms that occur between the host and the parasite are not fully understood, cathepsins and other proteases present in excretory/secretory extracts of *F. hepatica* have been related to tissue penetration (Robinson *et al*., 2009; Meemon and Sobhon, 2015). Migration through host tissues and establishment have also been linked with the interaction between different groups of parasites and the final enzyme of the fibrinolytic system, the protease of serines, plasmin (González-Miguel *et al*., 2016).

The fibrinolytic system comprises the mechanisms responsible for lysing the fibrin clot once formed and maintain, together with the coagulation cascade and platelets, a correct hemostatic balance in the circulatory system (Castellino and Ploplis, 2005; Chapin and Hajjar, 2015). From a molecular point of view, fibrinolysis is mediated by the conversion of plasminogen to its proteolytically active form, plasmin. This process is strictly regulated by binding of plasminogen to receptors through its five kringle domains, which have an affinity for lysine residues and for their activators, namely tissue plasminogen activator (tPA) and urokinase (uPA) (Cesarman-Maus and Hajjar, 2005). Plasmin as an active protease is capable of efficiently degrading the formed clot, but also different components of the extracellular matrix and connective tissue. This allows to relate the recruitment of this enzyme by blood and/or tissue-parasites with their dissemination, establishment and survival in host tissues (Figuera *et al*., 2013; González-Miguel *et al*., 2016).

From the point of view of fascioliasis, the interest of this fibrinolytic system and the tPA mainly, but not only, relies on the multifunctional roles of plasmin in inflammation (Li, 2005; Syrovets *et al*., 2012; Lin and Hu, 2014) including central nervous system affection (Gur-Wahnon *et al*., 2013) and blood-brain barrier permeability (Yepes *et al*., 2003; Michalski *et al*., 2012; Niego *et al*., 2012; Jin *et al*., 2014; Niego and Medcalf, 2014; Marcos-Contreras *et al*., 2016; Suzuki *et al*., 2016) including neurological effects (Fanne *et al*., 2010; Dong *et al*., 2016).

The objective of the present work is to study the interaction between an excretory/secretory extract of *F. hepatica* worms and the fibrinolytic system of its host by analysing their pro-fibrinolytic potential and to identify by proteomic techniques the antigens responsible for this interaction. The baseline furnished by the results obtained is analysed within the context of the complexity and heterogeneity of the clinical pictures shown by fascioliasis patients presenting with the aforementioned disorders. A proposal is finally exposed which for the first time allows to explain the different clinical situations reported in such fascioliasis patients.

## Materials and methods

### Materials

A *F. hepatica* isolate and lymnaeid snail vectors from a human fascioliasis endemic area were used. Metacercariae were obtained from experimentally infected *Galba truncatula* snails at the Department of Parasitology, University of Valencia, stored in freshwater at 4° C until required and administered to male rats after checking viability by use of the refractile appearance of the excretory granules as a criterion. *Galba truncatula* that shed the cercariae that gave rise to the metacercariae were from a laboratory-reared strain (in Heraeus-Vötsch HPS 1500 and HPS 500 climatic chambers; experimental conditions: temperature, 20° C; photoperiod, 12 h of light and 12 h of darkness; relative humidity, 90%). These snails were, in turn, infected by one miracidium (Mas-Coma *et al*., 2001). The Wistar rat was used as an animal model because of its characteristics similar to the human host from the points of view of parasite development (Valero *et al*., 2002) and host pathogenicity (Valero *et al*., 2003, 2006, 2008). Five male Wistar rats (80–100 g) were infected with 20 metacercariae/rat, by use of an orogastric syringe. A balanced commercial rodent diet (Panlab Chow A04) and water were provided *ad libitum*. Animal care, animal health, body condition and well being were assessed on a weekly basis by means of checking their body weight and the appearance of the fur. Infected animals presented a lower body weight than negative controls at the end of the experiment. No mortality occurred. At 10 weeks post-infection, animals were humanely euthanized with an overdose of the anesthetic (IsoFlo^®^; Dr Esteve SA, Barcelona, Spain). The worms were obtained in each rat by necropsy.

### Collection of excretory/secretory extract of proteins from *F. hepatica* adult worms

To prepare excretory/secretory products from *F. hepatica* adults (FhES), liver flukes were collected from Wistar rats. Liver flukes were cultured at concentrations of 1 worm mL^−1^ of medium for 12 h at 37° C. The medium was collected and centrifuged. After initial centrifugation at low speed to remove larger particles, the supernatant fraction was centrifuged at 15 000 ***g*** for 30 min at 4° C, and the supernatant was collected and concentrated to 1 mg mL^−1^ using an ultrafiltration membrane (YM-3, Amicon).

### Plasminogen-binding assay

In order to determine whether the FhES extract has the ability to bind plasminogen, an enzyme-linked immunosorbent assay (ELISA) was performed (González-Miguel *et al*., 2012). In brief, multiwell microplates (Costar) were coated with 1 µg per well of FhES extract diluted in carbonate buffer, pH 9.6, overnight at 4 °C. The wells were blocked with 1% BSA in PBS and incubated successively with increasing amounts (from 0 µg to 3 µg) of human plasminogen (Acris Antibodies), with a sheep anti-human plasminogen IgG (Acris Antibodies) at 1:2000 dilution and then with a peroxidase-conjugated donkey anti-sheep IgG (Sigma) at 1:4000 dilution. All incubations were performed for 1 h at 37 °C and between each step washed three times with PBS wash buffer (PBS containing 0.05% Tween_20_). Ortho-phenylene-diamine was used as a chromogen. Optical densities (OD) were measured at 492 nm in an Easy Reader (Bio-Rad). In parallel, competition assays were performed by including 50 mm of the lysine analogue ε-aminocaproic acid (εACA) during plasminogen incubation. Some wells coated with BSA only were used as negative controls.

### Plasmin generation assay

Plasminogen activation and subsequent fibrinolysis was analysed in a test volume of 100 µL by measuring the amidolytic activity of generated plasmin (González-Miguel *et al*., 2012). In each well, 2 µg of human plasminogen (Acris Antibodies) were incubated in PBS with 3 µg of the chromogenic substrate D-Val-Leu-Lys 4-nitroanilide dihydrochloride (Sigma) in the presence of 1 µg of FhES. Activation of plasminogen was initiated by the addition of 15 ng of tPA (Sigma). In parallel, plasmin generation was also measured in the absence of t-PA, to observe the ability of the FhES extract proteins of activating plasminogen on their own. Plates were incubated at 37° C for 1 h and the hydrolysis of the chromogenic substrate was analysed by measuring absorbance at 405 nm in an Easy Reader (Bio-Rad).

### Two-dimensional electrophoresis of FhES extract

FhES extract was subjected to two-dimensional electrophoresis as described before by us for the antigenic extracts from other parasites (Oleaga *et al*., 2009; González-Miguel *et al*., 2014). Briefly, FhES extract was purified with the ReadyPrep 2-D Cleanup Kit (Bio-Rad) following the manufacturer's recommendation and resuspended in rehydration buffer 2-D (7 M urea, 2 M thiourea, 4% 3-[(3-cholamidopropyl) dimethylammonio]-1-propanesulfonate (CHAPS)). The samples were divided into 125 µL aliquots (containing 60 µg of protein) and stored at −20° C until use. When they were used FhES aliquots were supplemented with ampholytes and 5 M DTT, incubated and centrifuged to remove all particulate material and then applied to 7-cm IPG strips (Bio-Rad) with linear pH ranges of 3–10, 5–8 and 7–10, using a Protean isoelectric focusing (IEF) Cell (Bio-Rad) for IEF. After IEF, strips were reduced and alkylated, and second dimension separation was done in 12% acrylamide gels. Gels were then silver stained with the PlusOne Silver Staining Kit (GE Healthcare), with the Sypro Ruby fluorescent dye (Bio-Rad) for quantitative analysis or transferred to nitrocellulose membranes for their immunoblot analysis. The 2-D images were scanned with the GS-800 Densitometer (Bio-Rad) and analysed with the Quantity One Software v.4.6.5 (Bio-Rad).

### Plasminogen ligand blotting assay

To determine which proteins of FhES extract bind plasminogen, they were electrotransferred from 2D gels to nitrocellulose membranes at 20 V for 30 min using a Trans-Blot SD Semi-Dry Transfer cell (Bio-Rad). Blots were blocked with 2% BSA in PBS wash buffer, for 1 h at room temperature. FhES membranes were incubated overnight at 4 °C with 10 µg mL^−1^ of human plasminogen. Then, the blots were incubated with a sheep anti-human plasminogen IgG (Acris Antibodies) at 1:1000 dilution and with a peroxidase-conjugated donkey anti-sheep IgG (Sigma) at 1:2000 dilution. These incubations were performed at 37° C for 90 min with shaking and between each step washed three times with washing buffer for 5 min per wash. Protein bands were revealed with 4-chloro naphthol. Negative controls were also used in which the plasminogen had been omitted. Membranes were digitized with the scanner GS-800 Densitometer (Bio-Rad) using the Quantity One Software v.4.6.5 (Bio-Rad). Matching of 2-D gels with the homologous Western blot to identify plasminogen-binding proteins, the assignment of molecular weights (MW) and isoelectric points (pI) of each protein were analysed using the PDQuest Software v.8.0.1 (Bio-Rad). All assays were performed in triplicate to assess the reproducibility of the spot pattern.

### Mass spectrometry (MS) and protein identification

The spots containing plasminogen-binding proteins were excised manually from the gels and sent to the proteomics facility of SCSIE University of Valencia (Valencia, Spain) for MS analysis. Samples were digested with sequencing grade trypsin (Promega) as previously described (Shevchenko *et al*., 1996). The digestion was stopped with 1% trifluoroacetic acid and the digested peptides were concentrated. A BSA plug was analysed in the same way to control the digestion process.

The resulting mixtures were analysed in a 5800 MALDI TOF/TOF (ABSciex) in positive reflector mode. Five of the most intense precursors (according to the threshold criteria: minimum signal-to-noise: 10, minimum cluster area: 500, maximum precursor gap: 200 ppm, maximum fraction gap: 4) were selected for every position for the MS/MS analysis. And, MS/MS data were acquired using the default 1 kV MS/MS method. The MS-MS/MS information was sent to MASCOT *via* the Protein Pilot (ABSciex). Database search was performed on the NCBI database. Searches were done with tryptic specificity allowing one missed cleavage and a tolerance on the mass measurement of 100 ppm in MS mode and 0.8 Da in MS/MS mode. Carbamidomethylation of Cys was used as a fixed modification and oxidation of Met and deamidation of Asn and Gln as variable modifications.

When a positive identification was not achieved, spots were analysed by liquid chromatography and tandem MS (LC–MS/MS). In this case, 5 µL of every sample was loaded onto a trap column (NanoLC Column, 3μ C18-CL, 350 um × 0.5 mm; Eksigent) and desalted with 0.1% trifluoroacetic acid at 3μL per min during 5 min. The peptides were then loaded onto an analytical column (LC Column, 3 µ C18-CL, 75 um × 12 cm, Nikkyo) equilibrated in 5% acetonitrile and 0.1% formic acid. Elution was carried out with a linear 5–45% gradient of solvent B (95% acetonitrile, 0.1% formic acid) at a flow rate of 300 nL per min. Peptides were analysed in a mass spectrometer nanoESI-Q-TOF (5600 TripleTOF, ABSciex). The tripleTOF was operated in information-dependent acquisition mode, in which a 0.25-s TOF MS scan from 350–1250 m z^−1^, was performed, followed by 0.05-s product ion scans from 100 to 1500 m z^−1^ on the 50 most intense 2–5 charged ions. ProteinPilot default parameters were used to generate peak list directly from 5600 TripleTOF wiff files. The Paragon algorithm of ProteinPilot was used to search NCBI protein database with the following parameters: trypsin specificity, iodoacetamide cys-alkylation and the search effort set to rapid. To avoid using the same spectral evidence in more than one protein, the identified proteins are grouped based on MS/MS spectra by the Protein-Pilot Progroup algorithm. Thus, proteins sharing MS/MS spectra are grouped, regardless of the peptide sequence assigned. The protein within each group that can explain more spectral data with confidence is shown as the primary protein of the group. Only the proteins of the group for which there is individual evidence (unique peptides with enough confidence) are also listed, usually toward the end of the protein list. Finally, the mascot generic files obtained for every spot by Protein Pilot were sent to MASCOT. Database search was performed on the NCBI TREMATODA-ESTs database. The molecular function and biological processes of the identified proteins were assigned according to the gene ontology database (http://www.geneontology.org) and the Swiss-Prot/UniProt database (http://beta.uniprot.org).

### Statistical analysis

The results from the plasminogen-binding assay and plasminogen activation assay were analysed with the Student's *t*-test. The results were expressed as the mean ± s.e.m. of at least three independent experiments. In all experiments, a significant difference was defined as a *P* value of <0.05 for a confidence level of 95%.

## Results

### Proteins of FhES extract bind plasminogen

**Fig. 1.**
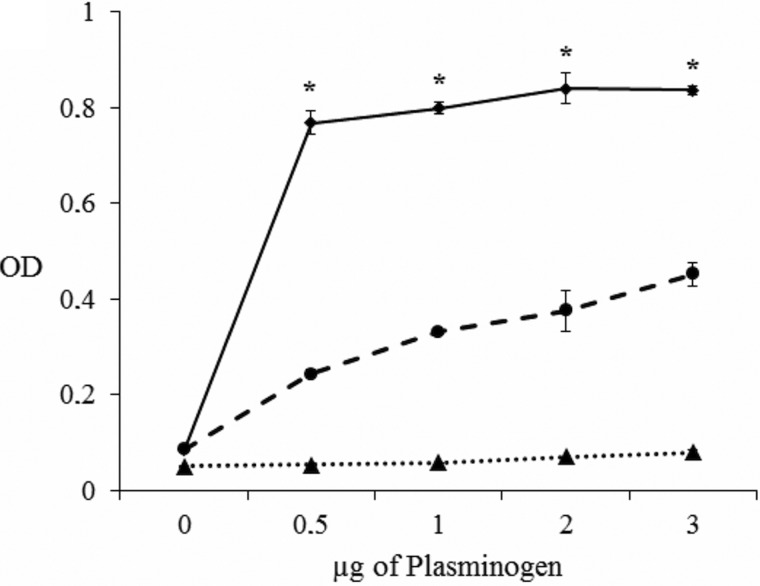
Plasminogen binding to 1 µg of FhES extract measured over a range of plasminogen amounts using a microtitre plate method: (■) Incubation with increasing amounts of plasminogen, 0–3 µg. (●) Competition assay with 50 mm εACA included during plasminogen incubation. (▲) Negative control consisted of wells coated only with BSA. Each point is the mean of three replicates ± s.d. The asterisk (*) designates significant (*P* < 0.05) differences.

The binding level of plasminogen to FhES extract was assessed by ELISA (Fig. 1). Analyses showed that proteins of FhES extract bind plasminogen and that this binding is directly proportional to the amount of plasminogen. The negative control consisting of wells coated only with BSA showed some non-specific binding activity, but always with values significantly lower than those obtained by FhES (*P* < 0.05). To determine whether or not lysine residues are involved in binding, a competition experiment including 50 mm εACA was carried out. In this case, the binding was inhibited about 60% resulting in slightly higher optical densities than the negative control (Fig. 1).

### Proteins of FhES extract enhance the activation of plasminogen by tPA

**Fig. 2.**
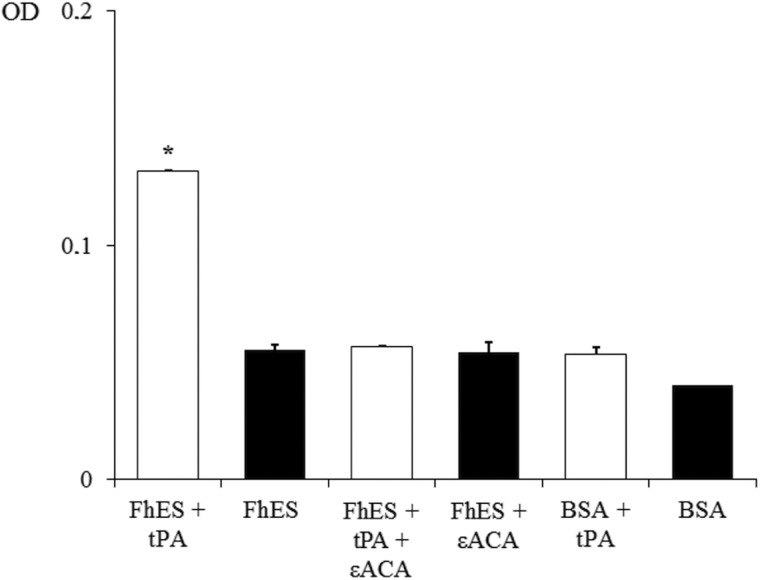
Plasminogen activation and plasmin generation by FhES extract of *Fasciola hepatica*: (□) 15 ng of t-PA was added to mixtures containing 2 µg of human plasminogen, 3 µg of D-Val-Leu-Lys 4-nitroanilide dihydrochloride (Sigma) and 1 µg of FhES extract (or BSA as negative control) in the presence or absence of 50 mm of εACA in a test volume of 100 µL. (■) No t-PA was added to reaction mixtures. Each point is the mean of three replicates ± s.d. The asterisk (*) designates significant (*P* < 0.05) differences.

In order to assess the ability of FhES extract to activate plasminogen and generate plasmin on their own, the amidolytic activity of plasmin generated in the presence or absence of tPA was measured. Negative controls replacing FhES for BSA or tPA were also used. Figure 2 shows the capacity of proteins of FhES to stimulate plasmin generation by tPA obtaining optical densities significantly higher than the negative controls (*P* < 0.05). Furthermore, this effect is inhibited by 50 mm εACA, indicating the involvement of lysine residues in the process. Plasminogen-activation did not occur in the absence of tPA.

### Separation of proteins of FhES extract by two-dimensional electrophoresis

**Fig. 3.**
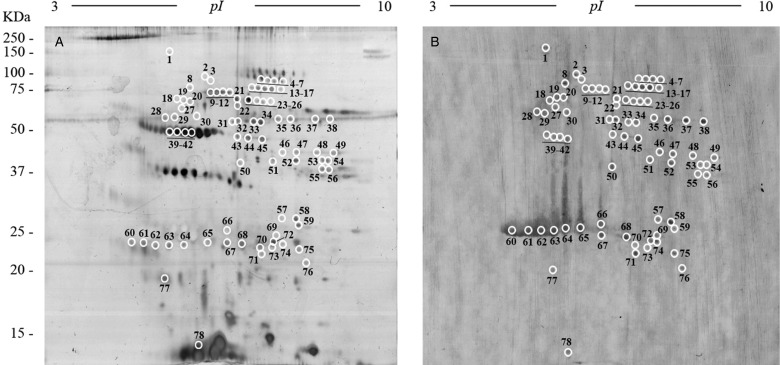
Two-dimensional electrophoresis of the FhES and ligand blotting with plasminogen: (A) Representative 2-DE of 60 µg of the FhES extract from adult *F. hepatica* flukes. The gels were in the 3–10 pH range, 12% polyacrylamide and silver-stained. (B) Ligand blotting assay to determine which proteins of FhES extract bind plasminogen. The plasminogen-binding spots revealed are circled and numbered. Reference molecular masses are indicated on the left.

To obtain an overall view of all the proteins of the FhES extract, it was first electrofocused using 3–10 linear immobilized pH gradient strips. Silver nitrate staining of these 2-D gels revealed 341 spots in the excretome of *F. hepatica* with pIs between 5 and 9.7, and a broad range of MWs (10–150 kDa). Only 33 spots were observed with pI <5 (Fig. 3A).

In order to improve spot resolution and detection, once the spot MW and pI ranges were determined, the FhES extract was electrofocused in 5–8 and 7–10 IPG strips. With these new conditions, silver staining revealed only 168 spots, most of them (119) located between pH 5 and 8 (not shown). We proceeded to use linear pH ranges of 3–10 for IEF of the FhES extract in the following experiments, due to the loss of resolution by using narrower pH ranges.

### Identification of plasminogen-binding proteins

To identify plasminogen-binding proteins, a ligand blotting with plasminogen of 2D gels of 3–10 pH was performed. As shown in Fig. 3B, a total of 78 plasminogen-binding spots were revealed (22.87% for total spots revealed in the excretome of *F. hepatica*). All of them were resolved in a range of MWs and pIs between 20 and 90 kDa, and 4.8 and 8.7, respectively. In the control blots, in which plasminogen incubation was omitted, the anti-plasminogen antibody did not reveal any spots (not shown).

The matching of spots revealed by ligand-blotting with their homologous in the silver-stained 2-D gels allowed us to select 47 representative plasminogen-binding spots of *F. hepatica*, which were manually excised from 2-D gels and submitted to analysis by MS.

Table 1 shows the identity of these proteins, the number of access to similar information available in the NCBI database and their MWs and pIs (theoretical and experimental). All of the spots were identified (*n* = 47) and corresponded to 16 different proteins. Between 1 and 6 isoforms of each protein were identified. Most of the identified spots corresponded to proteins of *F. hepatica* deposited in databases (38 of 47 spots). The 9 remaining spots corresponded to proteins from other trematodes (*Schistosoma haematobium*, *Schistosoma japonicum*, *Clonorchis sinensis* and *Opistorchis viverrini*). In addition, Table 1 shows the molecular function and biological processes in which the 16 proteins identified are involved. Among these, most were related to energy generation and metabolic pathways (6 proteins/16 spots), cell redox homeostasis or detoxification (5 proteins/13 spots) and to proteolysis (3 proteins/15 spots).

**Table 1.** Plasminogen-binding spots of the FhES extract identified by MALDI-TOF/TOF or LC–MS/MS

Spot number	Accesion code	Protein definition	Species	MW (kDa) theor/exp	pI theor/exp	Molecular function	Biological process
1	Q24940	Cathepsin L-like proteinase	*F. hepatica*	36.9/145.8	6.7/5.5	Cysteine-type endopeptidase activity	Proteolysis
5	CAA06158	Thiol-specific antioxidant protein	*F. hepatica*	21.7/93.6	6.3/7.3	Peroxiredoxin activity	Cell redox homeostasis
8	AAB41670	Secreted cathepsin L 1	*F. hepatica*	36.8/77.4	6.3/5.9	Cysteine-type peptidase activity	Proteolysis
11	AAP49831	Cathepsin L	*F. hepatica*	36.6/72.8	5.3/6.4	Cysteine-type peptidase activity	Proteolysis
15	CAA06158	Thiol-specific antioxidant protein	*F. hepatica*	21.7/75.4	6.3/7.4	Peroxiredoxin activity	Cell redox homeostasis
16	CAA06158	Thiol-specific antioxidant protein	*F. hepatica*	21.7/75.2	6.3/7.5	Peroxiredoxin activity	Cell redox homeostasis
19	AAP49831	Cathepsin L	*F. hepatica*	36.6/67.3	5.3/5.8	Cysteine-type peptidase activity	Proteolysis
21	Q27655	Enolase	*F. hepatica*	46.3/68.3	6.3/6.8	Phosphopyruvate hydratase activity	Glycolytic process
23	ACJ12894	Cathepsin L1D	*F. hepatica*	36.6/68.3	6.3/7.0	Cysteine-type peptidase activity	proteolysis
28	CAA12644	Protein disulphide isomerase	*F. hepatica*	55.2/57.7	5.1/5.3	Protein disulfide isomerase activity	Cell redox homeostasis
31	CAM96615	Thioredoxin-glutathione reductase	*F. hepatica*	66.0/55.6	6.5/6.7	Oxidoreductase activity	Cell redox homeostasis
33	Q27655	Enolase	*F. hepatica*	46.3/55.7	6.3/7.1	Phosphopyruvate hydratase activity	Glycolytic process
34	XP_012794303	Dihydrolipoyl dehydrogenase	*S. haematobium*	48.9/55.0	6.6/7.3	Dihydrolipoyl dehydrogenase activity	Cell redox homeostasis
35	CAA06158	Thiol-specific antioxidant protein	*F. hepatica*	21.7/57.0	6.3/7.9	Peroxiredoxin activity	Cell redox homeostasis
36	XP_012802048	Glutamate dehydrogenase	*S. haematobium*	62.1/57.1	8.8/8.1	Oxidoreductase activity	Amino acid metabolic process
37	XP_012802048	Glutamate dehydrogenase	*S. haematobium*	62.1/57.1	8.8/8.4	Oxidoreductase activity	Amino acid metabolic process
38	XP_009163890	Hypothetical protein T265_01567	*O. viverrini*	59.0/57.3	8.5/9.1	Oxidoreductase activity	Amino acid metabolic process
39	AAP49831	Cathepsin L	*F. hepatica*	36.6/51.1	5.3/5.4	Cysteine-type peptidase activity	Proteolysis
40	ACJ12894	Cathepsin L1D	*F. hepatica*	36.6/50.7	6.3/5.7	Cysteine-type peptidase activity	Proteolysis
42	ACJ12894	Cathepsin L1D	*F. hepatica*	36.6/49.9	6.3/6.1	Cysteine-type peptidase activity	Proteolysis
44	Q27655	Enolase	*F. hepatica*	46.3/48.4	6.3/7.0	Phosphopyruvate hydratase activity	Glycolytic process
45	Q27656	Enolase	*F. hepatica*	46.3/48.0	6.3/7.3	Phosphopyruvate hydratase activity	Glycolytic process
47	Q27657	Enolase	*F. hepatica*	46.3/43.7	6.3/7.8	Phosphopyruvate hydratase activity	Glycolytic process
48	AAW26182	SJCHGC01945 protein	*S. japonicum*	48.4/43.5	8.6/8.4	–	–
49	AAW26182	SJCHGC01945 protein	*S. japonicum*	48.4/43.4	8.6/8.8	–	–
50	Q27657	Enolase	*F. hepatica*	46.3/40.3	6.3/6.9	Phosphopyruvate hydratase activity	Glycolytic process
51	GAA28648	Fructose-bisphosphate aldolase	*C. sinensis*	39.8/41.4	8.7/7.5	Fructose-bisphosphate aldolase activity	Glycolytic process
52	GAA28648	Fructose-bisphosphate aldolase	*C. sinensis*	39.8/41.1	8.7/8.0	Fructose-bisphosphate aldolase activity	Glycolytic process
53	GAA28648	Fructose-bisphosphate aldolase	*C. sinensis*	39.8/41.2	8.7/8.5	Fructose-bisphosphate aldolase activity	Glycolytic process
55	AAG23287	GAPDH	*F. hepatica*	23.4/38.6	7.1/8.5	Oxidoreductase activity	Glycolytic process
58	AGJ83762	Triose phosphate isomerase	*F. hepatica*	27.8/27.9	8.1/8.0	Triose-phosphate isomerase activity	Gluconeogenesis
59	AGJ83762	Triose phosphate isomerase	*F. hepatica*	27.8/26.8	8.1/8.0	Triose-phosphate isomerase activity	Gluconeogenesis
60	P56598	Glutathione S-transferase 47	*F. hepatica*	25.7/24.1	6.6/4.7	Glutathione transferase activity	Metabolic process
61	Q24940	Cathepsin L-like protease	*F. hepatica*	36.9/23.4	6.7/5.0	Cysteine-type endopeptidase activity	Proteolysis
62	AAP49831	Cathepsin L	*F. hepatica*	36.6/23.5	5.3/5.2	Cysteine-type peptidase activity	Proteolysis
63	ACJ12894	Cathepsin L1D	*F. hepatica*	36.6/23.8	6.3/5.5	Cysteine-type peptidase activity	Proteolysis
64	ACJ12894	Cathepsin L1D	*F. hepatica*	36.6/23.8	6.3/5.8	Cysteine-type peptidase activity	Proteolysis
65	A8W7J0	Procathepsin L1	*F. hepatica*	35.7/24.2	5.6/6.3	Cysteine-type peptidase activity	Proteolysis
66	A8W7J0	Procathepsin L1	*F. hepatica*	35.7/26.1	5.6/6.6	Cysteine-type peptidase activity	Proteolysis
67	ACJ12894	Cathepsin L1D	*F. hepatica*	36.6/23.8	6.3/6.6	Cysteine-type peptidase activity	Proteolysis
68	P31670	Glutathione S-transferase	*F. hepatica*	25.4/24.0	6.5/6.9	Glutathione transferase activity	Detoxification
70	P31670	Glutathione S-transferase	*F. hepatica*	25.4/23.9	6.5/7.2	Glutathione transferase activity	Detoxification
72	P31670	Glutathione S-transferase	*F. hepatica*	25.4/24.1	6.5/7.5	Glutathione transferase activity	Detoxification
75	P31670	Glutathione S-transferase	*F. hepatica*	25.4/23.0	6.5/8.1	Glutathione transferase activity	Detoxification
76	CAA06158	Thiol-specific antioxidant protein	*F. hepatica*	21.7/21.3	6.3/8.2	Peroxiredoxin activity	Cell redox homeostasis
77	CAA06158	Thiol-specific antioxidant protein	*F. hepatica*	21.7/19.6	6.3/5.4	Peroxiredoxin activity	Cell redox homeostasis
78	Q7M4G0	Fatty acid-binding protein Fh15	*F. hepatica*	14.7/14.6	5.9/6.1	Lipid binding	Transport

Exp, experimental; theo, theoretical.

### Quantitative analysis of proteins of FhES extract by Sypro Ruby fluorescent dye

**Fig. 4.**
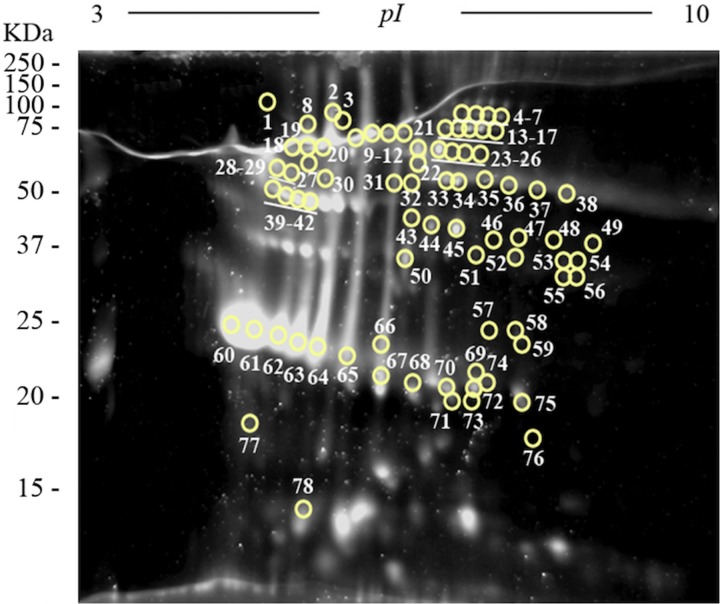
Quantitative analyses of the FhES extract by Sypro Ruby florescent dye: Representative 2-DE of 60 µg of the FhES extract from adult *F. hepatica* flukes. The gels were in the 3–10 pH range, 12% polyacrylamide and Sypro Ruby-stained. Note density of proteins as the appearance of clear spots on a dark background, which is directly proportional to the amount of each protein into the gel. The plasminogen-binding spots revealed on the ligand blotting assay are circled and numbered.

Two-dimensional electrophoresis gels were stained with Sypro Ruby fluorescent dye in order to perform a quantitative analysis using the PDQuest Software v.8.0.1 (Bio-Rad) and to select the most abundant plasminogen-binding spots in the excretome of *F. hepatica*. The most abundant proteins were resolved in a narrow range of MWs and pIs (between 22 and 68 KDa, and 4.7 and 6, respectively) (Fig. 4). The ten plasminogen-binding spots with the higher optical density after Sypro Ruby staining and, therefore, relatively more abundant in the FhES extract were spots number 23, 40, 42, 45, 60–64 and 67 and corresponded to different isoforms of cathepsin L, cathepsin L1D, enolase y glutathione S-transferase (see Table 1).

## Discussion

### Plasminogen-binding proteins in the *Fasciola hepatica* secretome/excretome

Plasmin produced by the activation of plasminogen is a broad-spectrum enzyme whose proteolytic activity, in addition to degrading the fibrin clot, has been linked to the lysis of extracytoplasmic matrices (Vassalli *et al*., 1991). This fact has allowed to relate the secretion of plasminogen receptors carried out by different groups of parasites with their dissemination, establishment and survival in host tissues (González-Miguel *et al*., 2016). Regarding trematodes, their interaction with the fibrinolytic system of their host has been widely studied in schistosomes (Mebius *et al*., 2013), while several proteins with plasminogen-binding capacity, among them an enolase in the excretory/secretory products of *F. hepatica* (Bernal *et al*., 2004), have been identified in *S. mansoni*, *S. japonicum*, *S. bovis*, *Clonorchis sinensis* or *Echinostoma caproni* (Marcilla *et al*., 2007; Ramajo-Hernández *et al*., 2007; de la Torre-Escudero *et al*., 2010, 2012; Yang *et al*., 2010; Wang *et al*., 2011; He *et al*., 2014; Hu *et al*., 2014; Figueiredo *et al*., 2015).

In the present work, we demonstrate that the FhES extract binds plasminogen and is capable of stimulating the generation of plasmin in an *in vitro* system. Activation of plasminogen only occurs in the presence of the fibrinolytic activator tPA, which is consistent with previously obtained results with antigens secreted by other parasites (Figuera *et al*., 2013). In addition, competition assays performed with ε-ACA acid reveal the involvement of the lysine residues of the FhES proteins in the process. This has particular relevance since the involvement of this type of residues has been described in the interaction with plasminogen (Rijken and Lijnen, 2009).

The proteomic analysis of the FhES extract together with the mass spectrometry allowed the identification of 47 plasminogen-binding spots, which corresponded to 16 different proteins. The large percentage of identification was made possible by the existence of available information on *F. hepatica* proteins in the databases, as a result of the recent publication of the parasite genome (Cwiklinski *et al*., 2015). Among them, the glycolytic enzymes enolase, glyceraldehyde phosphate-dehydrogenase and fructose bisphosphate-aldolase have been extensively studied for their role as plasminogen-binding proteins in a large number of pathogenic species of fungi, bacteria and parasites (Stie *et al*., 2009; de la Paz Santangelo *et al*., 2011; Bhattacharya *et al*., 2012; Funk *et al*., 2016; González-Miguel *et al*., 2016). Among the remaining metabolic proteins identified, triose phosphate isomerase had previously been linked to the fibrinolytic pathway (Marcilla *et al*., 2007; Furuya and Ikeda, 2011), while others have been identified as plasminogen-binding proteins for the first time: glutamate dehydrogenase and hypothetical protein T265_01567.

Cathepsins are also described for the first time as plasminogen receptors in pathogens (15 isoforms identified). Cathepsins are a large family of cysteine endopeptidases abundantly expressed in the secretions of *F. hepatica* (McVeigh *et al*., 2012). Their important role in such important processes as host haemoglobin digestion for nutrition, invasion or migration justify that they have been used as therapeutic targets in vaccine trials (Meemon and Sobhon, 2015). In addition, the expression associated with *Fasciola* development of these proteases has been correlated with the passage of the parasite through host tissues (Stack *et al*., 2011).

Another widely represented group (13 isoforms, 5 proteins) and whose interaction with plasminogen is also documented here for the first time in parasites are proteins related to redox or detoxifying homeostasis (see Table 1). These molecules have been linked to mechanisms of immune evasion and to the protection of *F. hepatica* from the harmful reactive oxygen species released by host immune cells. In addition, the secretion of a wide variety of antioxidants depending on the migration of *Fasciola* through host tissues has been documented (Robinson *et al*., 2009).

The results of this work demonstrate that *F. hepatica* has a large repertoire of plasminogen-binding proteins, which could be translated as a redundant system that reveals the importance of plasminogen recruitment for this parasite. These antigens are also among the most abundant within the excretoma of *F. hepatica* as revealed by the quantitative analysis (see Fig. 4). The proteins identified have important functions related to energy generation or detoxification processes but may perform other important functions such as plasminogen binding when they are at the host/parasite interface. In this sense, recent studies have begun to study these moonlighting functions of parasitic glycolytic enzymes (such as enolase or GAPDH) when they are found in extracellular localization related to invasion or establishment in the host (Gómez-Arreaza *et al*., 2014).

The plasminogen-binding proteins identified in the present work are part of an extract of *F. hepatica* adult worms. However, many of them, such as enolase, GAPDH, FBAL, cathepsins L and L1, GST, thrioredoxin or Fh15, remain active from the immature stage of the parasite (Halton, 1997; McVeigh *et al*., 2012) so that the obtained results could allow to relate the fibrinolytic activation with the migration carried out by the juvenile forms of the parasite. Recruitment of plasminogen by the adult phases of *F. hepatica* and, therefore, the use of plasmin could be linked with its survival in bile ducts, as has already been related in other trematodes such as *C. sinensis* (Wang *et al*., 2011; He *et al*., 2014), or with the clot-prevention necessary for blood nutrition described in *F. hepatica* (Halton, 1997).

### Baseline of neurological, meningeal and ocular disorders in fascioliasis patients

In patients showing infection by *Fasciola* in the liver, serious neurological, meningeal and ocular symptoms have been reported, such as limb and facial paralysis including hemiplegia, paresis up to even tetraparesis, impressive walking problems and movement disorders, paraesthesia, speech disorders, loss of senses, convulsions, epilepsia and coma, confusion, disorientation and amnesia, visual hallucinations, extremely intense nystagmus, or permanent blindness, among many others (Mas-Coma *et al*., 2014*b*). Such patients should not be confused with others presenting similar disorders due to neurofascioliasis or intracranial infection and ophthalmofascioliasis or direct affection of the eye by migrating ectopic flukes. The latter two ectopic infections appear to be rare (Mas-Coma *et al*., 2014*b*).

A baseline obtained when comparing the clinical pictures in these neurological patients showing infection by *Fasciola* in the liver offers crucial information. Such disorders have been reported in patients of both sexes, all age groups and whether at the acute phase (sometimes even very early within the first symptoms) or in the chronic or advanced chronic phase (even several years after infection). Moreover, such neurological manifestations have been described in patients infected by both species of *Fasciola* and from throughout the world. This indicates that there is no relationship with *Fasciola* species nor with geographical strains of the liver flukes (Mas-Coma *et al*., 2014*b*).

The capacity of flukes in the liver to cause the aforementioned disorders at distance means their excretory/secretory products to be able to alter the blood-brain barrier. Surprisingly, in the patients affected by such neurological disorders in whom the fluke burden could be assessed, infection was by a very low number of flukes in the liver, namely between 1 and 12 flukes per subject suffering major neurological symptoms, with an exception of a neurological fatal case in whom 26 flukes were found (Mas-Coma *et al*., 2014*b*). Consequently, *a priori* it does not seem to be a direct relationship between burden and capacity to give rise to neurological disorders (Mas-Coma *et al*., 2014*b*), suggesting that individual host susceptibility may perhaps underlie the appearance of such disorders in given patients. Markers have already proved to be useful in human fascioliasis (Valero *et al*., 2016) and the availability of indicators allowing for the detection of patient susceptibility to suffer from such neurological disorders would, therefore, be welcome.

### Blood-brain barrier leakage in Fasciola infection

A study in the laboratory rat model demonstrated that (i) *F. hepatica* infection in the liver was able to impact on remote tissues, (ii) an altered neural nucleotide signature was one of the strongest metabolic responses in infected rats and (iii) the perturbed immunological function suggested by the altered nucleotide levels in the brains of *F. hepatica*-infected animals proved the neural effects were exclusive of *Fasciola* when compared with other trematodes as *S. mansoni* and *E. caproni* (Saric *et al*., 2010). Significant perturbations of the nucleotide balance in the brain, together with an increase of an important direct negative regulator of inflammatory cytokines in macrophages such as plasma IL-13, were found. This suggested a shift toward modulation of immune reactions by secretion of nucleotide-degrading enzymes to minimize inflammatory response, which may favour the co-existence of the parasite in the host. The pattern of differentiating metabolites in the neurochemical profiles was composed of a significant increase in the relative levels of inosine, tyrosine and phenylalanine. Conversely, the relative tissue concentrations of GPC, succinate, inosine mono-, di- and triphosphate, adenosine and adenosine mono-, di- and triphosphate were lower in the brains of infected laboratory rats (Saric *et al*., 2010).

In individual case reports, many mechanisms have been observed to accompany or suggested to be linked to the neurological disorders described in given patients presenting infection by *Fasciola* in the liver (Mas-Coma *et al*., 2014*b*): inflammation, vasculitis, arteritis, thrombosis; local and systemic responses; circulating immune complexes and complement system activation; autoimmune responses; hypersensivities, type III hypersensivity, Arthus reaction; impairment of the blood-brain barrier; hepatic encephalopathy and relationships with ammonia, sodium, manganese and others; hypereosinophilia-induced encephalopathy; proinflammatory cytokines, oxidative/nitrative stress; neurotoxins; substances derived from the damage, malfunction and failure of the liver; coinfections and sepsis. This suggests a large complexity and inter-relationships of immuno-allergic and physiopathogenic processes (Mas-Coma *et al*., 2014*b*). Interestingly, a neurological picture caused by *Fasciola* has also been found associated to a condition such as the antiphospholipid syndrome (Frances *et al*., 1994), whereas this in its turn was later also related to plasmin and the fibrinolytic system (Yang *et al*., 2004).

We here demonstrate that the excretory/secretory products of *Fasciola* may trigger a plasmin-dependent mechanism or pathway which increases blood-brain barrier permeability and cause neurological disorders.

*Fasciola hepatica* is able to interact with the fibrinolytic system of its host by secreting a large number of proteins with the ability to bind plasminogen and enhance the generation of plasmin. Worth mentioning is that most of these proteins (38 out of 47 spots) appear to be exclusive for *F. hepatica* so far, including a large number of cathepsin isoforms. The potential use of some of these antigens as novel targets for anthelmintic compounds and potential specific multi-antigenic vaccine candidates should be explored in the future.

The remaining few of the plasminogen-binding proteins identified in the present work are common to those that have been studied in other pathogens with very different host invasion strategies. This may be probably related to a process of evolutionary convergence, which determines the appearance of similar mechanisms to solve common problems.

### Fluke behaviour in the acute and chronic phases

*Fasciola* metacercariae infect humans after ingestion together with edible freshwater vegetables, drinking natural contaminated water, or terrestrial plants, fruits and tubercles washed with contaminated water (Mas-Coma *et al*., 2018). Metacercariae excyst in the small intestine within an hour after ingestion. A proportion of metacercariae die in the gastrointestinal tract and relatively few eventually penetrate the host's intestine wall, thus starting their active migration.

**Fig. 5.**
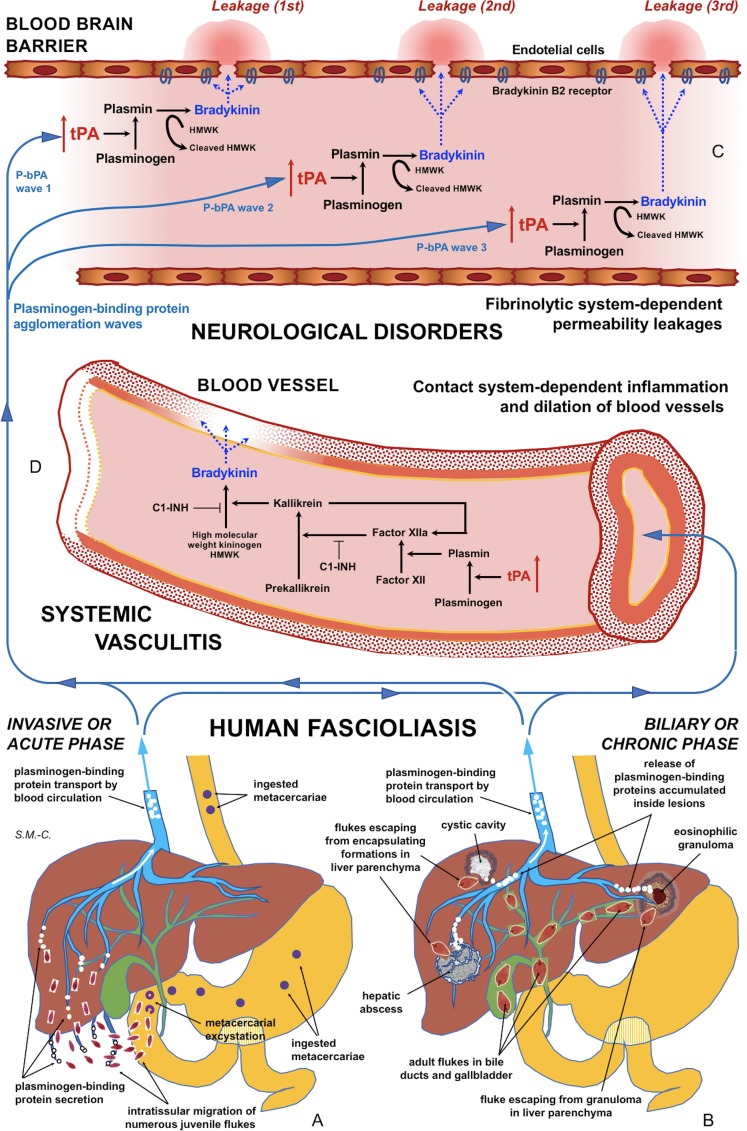
Schematic representation of the interaction between infecting *Fasciola* behaviour, fibrinolysis system alterations triggering subsequent blood-brain barrier leakages and contact system alterations inducing systemic vasculitis: (A) In the acute phase of the disease in cases of many simultaneously migrating, small-sized juvenile flukes after ingestion of numerous metacercariae. (B) In the chronic phase of the disease after the release of large amounts of accumulated excretory/secretory products following the breakage of encapsulating formations triggered by single worm tracks at different times. (C) Blood-brain barrier leakages subsequently occurring due to fibrinolytic system-dependent mechanism involving plasmin-dependent generation of bradykinin and subsequent activation of bradykinin B2 receptors, according to different plasminogen-binding protein agglomeration waves. (D) Inflammation and dilation of blood vessels due to contact system-dependent generation of the proinflammatory peptide bradykinin. Schema design and drawing by S. Mas-Coma.

This acute phase, also called invasive or migratory phase, comprises intraorganic migration until final penetration into the bile ducts where they become sexually mature (Fig. 5A). Fecundation, egg production and shedding by the mature worms, passive transport of the eggs with the bile up to the intestinal lumen and appearance of eggs in stools take at least 3–4 days. The pre-patent period includes from the ingestion of metacercariae to the first appearance of eggs in feces and takes at least 3–4 months in humans. This time may, therefore, be extrapolated to the duration of the migratory phase (shorter than the prepatent period in only 3–4 days) (Mas-Coma *et al*., 2014*b*). This phase is 1–2 weeks longer when in *F. gigantica* infection (Valero *et al*., 2016).

The initial penetration of the juvenile worms through the duodenum or jejunum walls may give rise to focal haemorrhages and inflammation. Juvenile worms appear in the abdominal cavity by about 2 h after ingestion. Afterwards, they migrate inside the peritoneum until contacting the liver. During this migration (Fig. 5A), the juvenile worms cause the extensive mechanical destruction of abdominal peritoneum and liver tissue causing intensive haemorrhagic lesions and immunological and inflammatory reactions, with localized or generalized toxic and allergic processes. Most reach the liver within 6 days after excystment. Juvenile flukes migrate through the liver parenchyma for 5–6 weeks, preferentially feeding directly on liver tissue. They finally penetrate into the bile ducts where they become sexually mature. Migratory parasites become sometimes trapped in the liver parenchyma and die without reaching the bile ducts, leaving cavities filled with necrotic debris and considerable liver areas may subsequently be replaced by scar tissue (Mas-Coma *et al*., 2014*b*). It has also been speculated that immature flukes may enter the blood stream and be carried to various parts of the body, or may reach the liver by travelling up the bile duct. In the case of failure to reach the biliary system of the liver, the immature flukes may die in the abdominal cavity and other parts of the body (Chen and Mott, 1990).

This chronic phase, also called biliary or obstructive phase (Fig. 5B), starts from the moment the flukes penetrate in the bile ducts, that is, 3–4 days before the first appearance of eggs in stools. Consequently, the presence of eggs in stools indicates the patient to be in that chronic phase. This phase may last for a very long time according to the adult worm life span of up to 13.5 years in humans (Mas-Coma *et al*., 2014*b*). The term ‘advanced chronic’ phase is used for long-lasting infections, when ingestion of metacercariae took place a long time ago, e.g. 1 or more years before patient diagnosis.

Once in their final hepatic microhabitat, the endocrine system of the host provides reliable signals that are part of the perceptual world of the blood-feeding *Fasciola* adult (Sukhdeo *et al*., 1988; Sukhdeo and Sukhdeo, 1989). Adult flukes in the bile ducts cause inflammation, hyperplasia of the epithelium and thickening and dilatation of duct and gall bladder walls.

Mature worms living inside the biliary canals may re-enter the liver parenchyma in different moments of the chronic phase (Han *et al*., 1999), return back to a biliary canal after a more or less short transit, or give rise to an encapsulating eosinophilic granuloma if staying in the parenchyma for a longer period (Fig. 5B). This may explain the findings of eggs of both *F. hepatica* and *F. gigantica* inside eosinophilic granulomas in the liver parenchyma (Arjona *et al*., 1995) or hepatic masses (Grange *et al*., 1974), although the possibility cannot be discarded that such worms were migrating flukes which never achieved a biliary canal at the end of their initial migration as juveniles and finished to mature inside the parenchyma. The finding of high numbers of *Fasciola* eggs in eosinophilic granulomas from peritoneum and intestinal wall suggest juvenile worms becoming encapsulated in granulomas in extrahepatic sites at the initial steps of their migratory pathway and subsequent capacity to mature and produce and shed eggs after selfing ‘*in situ*’ (Mohammadi Ghalehbin *et al*., 2010).

### Interactions between fluke behaviour and the fibrinolysis system

This capacity of *Fasciola* to secrete a large number of proteins with the ability to bind plasminogen and enhance the generation of plasmin may also underlie the leakage of the blood-brain barrier and subsequent induction of neurological disorders. There appear to be multifactorial and probably plasminogen-dependent effects of tPA on the blood-brain barrier (Niego *et al*., 2012; Niego and Medcalf, 2014). Chronic tPA overexpression has been seen to increase the permeability of the intact blood-brain barrier by a plasminogen-dependent mechanism involving plasmin-dependent generation of the proinflammatory peptide bradykinin and subsequent activation of bradykinin B2 receptors. It is possible that chronic *vs* acute increases in tPA levels in the circulation act by different mechanisms. It has been speculated that the initial blood-brain barrier leakage induced by bradykinin allows circulating tPA to enter the brain parenchyma, where it can exert further deleterious effects on blood-brain barrier integrity and glutamatergic transmission (Marcos-Contreras *et al*., 2016).

In patients in the invasive acute phase, presenting with neurological symptoms sometimes appearing suddenly and very early, the induction of neurological disorders is unavoidably caused by juvenile flukes migrating through different tissues. The rarity of these situations may be due to the infection by concomitant ingestion of numerous metacercariae giving rise to many juvenile flukes migrating simultaneously. An E/S product release sufficient for a blood-bain barrier leakage may happen in such unusual cases of a high number of small-sized, simultaneously migrating juvenile worms of around 250 µm in length (Mas-Coma *et al*., 2014*b*). This may explain the early appearance of neurological disorders even within the first disease symptoms (Fig. 5A).

Indeed, the very precocious and high activity of the migrating juveniles has already been assessed from the immunological point of view. By the time the newly excysted juveniles have penetrated the intestinal wall and entered the peritoneum, they have already initiated the immune events that will dominate throughout infection. So, these early-stage parasites secrete immunomodulatory molecules that influence the function of innate cells in the intestinal wall and peritoneal cavity. A systemic antigen-specific Th2 response is firmly established already at 7 days postinfection (Dalton *et al*., 2013).

In patients in the biliary chronic phase, even including neurological pictures appearing in the long term (many years after the infection), the induction of neurological disorders should be the consequence of other causes. In these patients, such a sufficient E/S product release may not depend on a massive *Fasciola* infection but on a very few, or perhaps single, large-sized adult worms. In the biliary canals, they have been reported to attain up to a length of 29 mm in *F. hepatica* and 52 mm in *F. gigantica* in cattle (Periago *et al*., 2006). In ectopic worms found in patients presenting with neurological manifestations, a maximum length of up to 8 mm has been reported (Mas-Coma *et al*., 2014*b*). The tracks followed by the worms and the subsequent physiopathological changes suffered by these tracks may allow for the accumulation of excretory/secretory products inside. These tracks give rise to numerous small holes and cavities and cause inflammation, nodular lesions, abscesses, cystic cavities, haemorrhages, necrosis, granulomas, fibrosis and formations described as liver mass. The breakage of such formations in the liver or ectopically in other neighbouring organs in the patient at different times in the chronic and advanced chronic phases may give rise to a simultaneous release of large amounts of accumulated excretory/secretory products (Fig. 5B).

Whole worms have been sometimes found inside these different types of lesions (Ghawabi *et al*., 1978; Ragab and Farag, 1978; Acosta-Ferreira *et al*., 1979; Park *et al*., 1984; Aroonroch *et al*., 2006; Kim *et al*., 2015). However, worm absence inside such formations is the most current observation reported from patients, indicating that these intratissular worms are able to escape from such host reactions. Mechanical tissue damage by the worms themselves during their tunnel boring, any accidental traumatism, or even perhaps their diffusion by permeability after tissue regeneration, may be at the origin of a quick, timely concentrated release of such E/S products and their access to the circulation.

Hyperfibrinolysis is the consequence of an imbalance between profibrinolytic factors, as tPA and uPA and antifibrinolytic factors, such as plasminogen activator inhibitors, a2-antiplasmin, or thrombin activatable fibrinolysis inhibitor (Shakur *et al*., 2010). The most evident pathological effect of hyperfibrinolysis is an increased bleeding (Roullet *et al*., 2015). The hyperfibrinolytic state was found to be the result of an increased level of profibrinolytic factors that is not (or not fully) compensated by an increased level of antifibrinolytic factors (Gando, 2015). This imbalance triggers the plasmin generation and leads to a severe bleeding tendency.

In fascioliasis, among the highly severe complications described in patients, there are reports on haemorrhages and haematomas (Lortat-Jacob *et al*., 1960; Piquet *et al*., 1986; Sauvage *et al*., 1996; Martinez *et al*., 2002; Loja *et al*., 2003; Rosas *et al*., 2008; Morales *et al*., 2009), as well as bleeding (Goodman *et al*., 1973), several of them fatal cases reported from children (Bannerman and Manzur, 1986; Acuña-Soto and Braun-Roth, 1987; Almendras-Jaramillo *et al*., 1997). In none of these cases were neurological manifestations reported.

However, in fascioliasis patients presenting with neurological disorders, bleeding has been only described in patients suffering from neurofascioliasis or intracranial infection by migrating ectopic flukes (Ying *et al*., 2007; Zhou *et al*., 2008), but in no case of patients presenting with neurological, meningeal, psychiatric or neuropsychic manifestations and/or ocular disorders caused at distance by flukes infecting the liver (Mas-Coma *et al*., 2014*b*). This appears to be similar to observations in a cohort of patients with ischemic stroke, among whom none presented hemorrhagic transformations (Marcos-Contreras *et al*., 2016).

This suggests that, in such fascioliasis patients, the balance between profibrinolytic and antifibrinolytic factors may not be altered or sufficiently modified at a systemic level. This does not mean, however, that a circulating plasmin agglomeration may be able to induce timely punctual leakages on the blood-brain barrier (Fig. 5C). Subsequent blood stream passes of such a (or other) plasmin agglomerations may be responsible for blood-brain barrier leakage on different sites, thus underlying triggering of different neurological manifestations. This would, moreover, allow to explain the puzzling polymorphisms and disconcerting multifocality of the neurological manifestations throughout time (Mas-Coma *et al*., 2014*b*).

### Inflammation and vasculitis in fascioliasis patients

Recent studies have linked an excess of plasmin result of a long-term pro-fibrinolytic parasite activity with pathogenic mechanisms that include cell proliferation and migration, inflammation and degradation of the extracellular matrix (González-Miguel *et al*., 2015, 2016). Consequently, the excess of plasmin generated, *a priori* beneficial to *F. hepatica*, could also be related to the typical inflammatory processes of fascioliasis.

tPA is a molecule with dual functions: (i) a serine protease, playing a pivotal role in the homeostasis of blood coagulation/fibrinolysis and extracellular matrix regulation; (ii) a cytokine, executing multiple actions by binding to its membrane receptors and triggering profound intracellular signaling events (Lin and Hu, 2014). tPA appears to have broad implication in the modulation of infiltration and inflammation in diverse organs. The increased inflammatory infiltration in most disease models is accompanied by the concomitant induction of tPA, suggesting that tPA may be a common endogenous factor that modulates inflammatory infiltration and response in multiple organ systems (Lin and Hu, 2014).

Of particular interest appear to be the role of tPA in cerebral inflammation (Wang *et al*., 1998; Zhang *et al*., 2007, 2009) and in liver inflammation (Higazi *et al*., 2008). The role of tPA has also been investigated in infection by pathogens. Results indicate that tPA may also play an important role in the modulation of innate immunity, which is fundamental in the host defense against pathogens (Lin and Hu, 2014).

In fascioliasis patients presenting with neurological, meningeal and/or ophthalmic manifestations, inflammatory cell reactions in the liver have been reported several times (Paraf *et al*., 1967; Lesecq *et al*., 1972; Aguirre Errasti *et al*., 1981; Berenger, 1984; Prociv *et al*., 1992). Moreover, in most recent patient reports the inflammation has been described to involve blood vessels giving rise to situations of systemic vasculitis (Oujamaa *et al*., 2003; Llanos *et al*., 2006; Málaga *et al*., 2012).

*Fasciola* may use the bile salts (phospholipids, cholesterol and bilirubin) to encapsulate its excretome/secretome in a similar way as micelles are formed. As it is a haematophagous worm, *Fasciola* might then release these vesicles to the circulatory system. These bile salt micelles act as a transport medium to carry the excretome/secretome to the surface of the cells of the blood-brain barrier. Here, in combination with inflammation-activated kallikrein, bradykinin cascade effects are triggered, including capillary leakage. Bradykinin effects only last for a very short time, which suggests that *F. hepatica* may release several micelles containing its excretome/secretome to the circulatory system.

The plasma kallikrein-kinin system, also known as contact system (Fig. 5D), is a group of plasma proteins that responds to the presence of pathophysiological materials and invasive pathogens, by means of a plasma protease cascade initiated by factor XII (FXII) that activates the proinflammatory kallikrein-kinin system and the procoagulant intrinsic coagulation pathway. The plasma contact system drives proinflammatory and procoagulant pathways and is composed of plasma proteases, substrates and inhibitors produced and secreted by the liver. It consists of coagulation factors XII (FXII) and XI (FXI), plasma prekallikrein (PPK), the non-enzymatic cofactor high molecular weight kininogen (HMWK) and C1 esterase inhibitor (C1INH). FXII becomes activated when it comes into contact with negatively charged surfaces and undergoes a conformational change, which generates small amounts of activated FXII (FXIIa). FXIIa cleaves PPK to form plasma kallikrein (PK), which reciprocally activates FXII and generates a positive feedback loop of FXII activation. PK subsequently digests HMWK to release the vasoactive peptide bradykinin (BK), a potent proinflammatory peptide and the end product of the kallikrein-kinin pathway. BK activates signaling pathways resulting in increased vasodilation in arteries and veins and vascular permeability (Long *et al*., 2016).

Contact system–dependent inflammation has been observed to be activated by multiple different pathogens, parasites included. Contact system activation is beneficial in clearing pathogens, but excessive BK formation may also have detrimental effects to the host (Long *et al*., 2016). In patients, abundant production of BK may induce vascular leak, exerting disadvantage effects and contributing to multiple organ failure. In severe sepsis patients, it has been observed that the kinin system is activated as exemplified by elevated plasma bradykinin and consumption of prekallikrein and FXII (Wu, 2015). In hantavirus infection, vascular leakage is caused by alterations of the endothelial barrier. Incubation of plasma proteins with hantavirus-infected endothelial cells results in HMWK cleavage, increase in enzymatic activities of FXIIa and kallikrein and liberation of bradykinin, leading to dramatic increases in endothelial cell permeability (Taylor *et al*., 2013).

There is experimental and clinical evidence that the fibrinolytic system triggers the activation of the contact system. *In vitro*, it has been demonstrated that plasmin can induce FXII activation. The importance of plasmin as a natural FXII activator is also supported by observations in patients in whom neurological affection is presumably mediated *via* plasmin-dependent bradykinin generation (Hofman *et al*., 2016).

The consideration that the contact system and fibrinolytic system are functionally linked (Fig. 5C and D) opens a field for the search of biomarkers useful to prevent neurological affection risk in fascioliasis patients. Contact system activation products in plasma may be valuable biomarkers as they may reflect recent bradykinin production, as it may be considered that bradykinin detection is complicated because it is only present in the circulation for a few seconds after it is released from HMWK, due to rapid degradation by kininases. Fibrinolytic biomarkers may also be helpful in identifying bradykinin-mediated neurological disorders (Hofman *et al*., 2016).

### Concluding remarks

Our results suggest that tPA overexpression due to the numerous plasminogen-binding proteins of the *Fasciola* excretome/secretome increases the permeability of the intact blood-brain barrier by a plasminogen-dependent mechanism. Blood stream passes of circulating plasmin agglomerations triggered by numerous concomitantly migrating juvenile flukes or by released inside-lesions concentrated E/S products shed by single or few adult worms may not only explain blood-brain barrier leakages and subsequent neurological manifestations in the acute and chronic phases, respectively, but also the puzzling polymorphisms and disconcerting multifocality of the neurological manifestations along the progressively changing clinical pictures in those patients (Fig. 5A–C).

This is, therefore, the first time that potential interactions between diverse mechanical parasitic situations and punctual non-imbalancing alterations of the fibrinolysis system in human infections are proposed that explain the large complexity, heterogeneity and timely variations of the neurological disorders in fascioliasis patients. Worth mentioning is that the explaining mechanisms proposed do not relate to the triggering of neurological disorders to individual subject susceptibility, neither in the acute nor in the chronic phase.

Reports of inflammation and vasculitis in fascioliasis patients presenting with neurological, meningeal and/or ophthalmic manifestations suggests that the fibrinolytic system triggers the activation of the contact system (Fig. 5A–D). The aforementioned proposal opens the door for the search of biomarkers for the distinguishing of fascioliasis patients with a risk to develop neurological affection, among both fibrinolysis system and contact system activation products in plasma. Moreover, antifibrinolytic treatments or B2 receptor antagonists should be investigated in laboratory animal models in the search of tools for preventing blood-brain barrier leakage which would potentially give rise to neurological manifestations in fascioliasis patients.

The blood-brain barrier leakage pathways linked to the fibrinolytic system and the contact system, triggered by *Fasciola* in neurologically affected patients, do not suggest a particular strategy by the liver fluke but merely a consequence of (i) infection by a high number of metacercariae when disorders appearing in the acute phase or by (ii) an uncommon liver parenchymal behaviour of the adult stage in the chronic phase. This appears to be different from other parasites also able to cause neurological disorders and in which matrix metalloproteinases (MMPs) have been found to be affected, modified or involved. These are species in which blood represents the final microhabitat or a crucial migration road and most of them having an evident tropism for the central nervous system. Examples are nematodiasis such as angiostrongyliasis or eosinophilic meningoencephalitis (Chiu and Lai, 2014) and several protozooses such as cerebral malaria (Polimeni and Prato, 2014), trypanosomiasis including sleeping sickness and Chagas disease, leishmaniasis and toxoplasmic encephalitis in immunocompromised hosts (Geurts *et al*., 2011). A mechanism by which parasite-derived products alter host expression of MMP and endogenous MMP inhibitors has only been described for hemozoin in malaria (Prato *et al*., 2011). Although a potential role of MMPs in blood-brain barrier leakage cannot *a priori* be disregarded, none of the characteristics of the host-parasite inter-relationships of a biliary trematode as *Fasciola* suggests that way.
