# Numerous *Fasciola* plasminogen-binding proteins may underlie blood-brain barrier leakage and explain neurological disorder complexity and heterogeneity in the acute and chronic phases of human fascioliasis – CORRIGENDUM

**DOI:** 10.1017/S0031182020000268

**Published:** 2020-05

**Authors:** J. Gonzalez-Miguel, M. A. Valero, M. Reguera-Gomez, C. Mas-Bargues, M. D. Bargues, F. Simon-Martin, S. Mas-Coma

The authors wish to apologise for an error in the Financial Support Section of the above article. The text:

Health Research Project No. PI16/00520, Subprograma Estatal de Generación de Conocimiento de la Acción Estratégica en Salud (AES), Plan Estatal de Investigación Científica y Técnica y de Innovación, ISCIII-MINECO, Madrid, Spain

should instead read:

Health Research Project No. PI16/00520, Subprograma Estatal de Generación de Conocimiento de la Acción Estratégica en Salud (AES) y Fondos FEDER, Plan Estatal de Investigación Científica y Técnica y de Innovación, ISCIII-MINECO, Madrid, Spain
